# Supplementary material for: Are carbon emissions trading and green financial instruments synergistic? -Comprehensive quantitative research based on content analysis
Source: PLoS One. 2024 Mar 7;19(3):e0298601. doi: 10.1371/journal.pone.0298601 (PMC10919601; doi:10.1371/journal.pone.0298601)
Supplement: S1 Data — (ZIP) [file pone.0298601.s001.zip › Data/地级市Z_F工作报告.zip!/║■─╧/│ñ╔│╩╨/└┤╫╘╣1⁄2╓┌║┼í╛┬φ┐╦╩2╛▌═°í┐_2019.docx]

各位代表：

　　现在，我代表市人民政府向大会报告工作，请予审议，并请各位政协委员和其他列席人员提出意见。

　　一、2018年工作回顾

　　2018年，我们在省委、省政府和市委的坚强领导下，全面贯彻落实习近平新时代中国特色社会主义思想和党的十九大精神，自觉践行新发展理念，深入实施创新引领开放崛起战略，着力建设现代化长沙和“三个中心”，砥砺奋进、攻坚克难，完成了市十五届人大二次会议确定的目标任务。全市地区生产总值增长8.5%（预计数，下同）；规模以上工业增加值增长7.6%；固定资产投资增长11.5%；社会消费品零售总额增长10%；一般公共预算收入增长10.09%；全体居民人均可支配收入增长8.6%。经济社会发展稳中有进、稳中趋优，民生实事全面完成，连续11年获评中国最具幸福感城市，老百姓和市场主体拥有更多获得感。

　　——老百姓呼吸的空气、饮用的水明显提质。蓝天保卫战初战告捷，全年空气质量优良天数278天，较上年增加16天，“长沙蓝”成为常态。“河长制”深入落实，“一河一策”全面实施，湘江长沙段水质达到Ⅲ类以上；雷锋水厂建成运营，一水厂改造、七水厂新建、望城水厂扩建加快推进，株树桥水库饮用水源一级保护严格落实，柏家洲水源地保护区居民全部搬迁，城市供水水质合格率100%；新增20万农村居民用上自来水。

　　——老百姓吃得舒心放心。米粉、豆制品实现集中生产、冷链配送，新增“明厨亮灶”餐饮单位5434家，整治违规夜市1.5万处，打造精品夜市示范点11个，国家食品安全示范城市创建通过省级验收，“舌尖上的安全”保障有力。

　　——老百姓住房保障切实加强。“反炒房”攻坚战取得阶段性胜利，持续保持房价“洼地”优势。完成棚户区改造2.29万户，棚改经验得到国务院通报表扬；改造农村危房5193户；13个城中村实现拆迁清零，5580户群众分到了新房；9121户居民新入住公租房，更多群众实现了安居梦。

　　——老百姓交通出行方便快捷。成功创建国家公交都市示范城市，公交分担率达54.2%。地铁4号线试运行，4个园区开通穿行巴士，20个社区公交直达地铁站。新建公共停车场137个、停车位2.68万个；新改建自行车道488.46公里，新建和提质改造人行道436.3公里；新建历史文化步道32.8公里；打通6条瓶颈路、断头路。城市“一卡通”正式发行，带上一张卡就能畅行长沙。

　　——老百姓安全感持续增强。扫黑除恶专项斗争、安保维稳“百日会战”成效明显，实现发案下降、破案上升、命案全破；安全生产形势平稳向好，事故起数、死亡人数大幅减少，人民群众生命财产安全得到有效保障。

　　——老百姓获得广泛实惠的民生福祉。“一圈两场三道”建设全力推进，建成15分钟生活圈241个，市民步行15分钟就可以享受到14类公共服务；新改扩建农贸市场76个、生鲜门店46个。新改扩建公办幼儿园33所；创建垃圾分类减量示范小区 20个；创建绿色示范村庄、示范社区100个；完成农村适龄妇女两癌免费检查7.04万例、孕产妇免费产前筛查6.5万例；为634名残疾儿童实施抢救性康复救助；建成群众性体育设施600处。

　　——长沙电力保障坚实有力。大力实施“630攻坚”，电网建设投资创历史新高，净增电力容量接近前5年总和，在725万千瓦历史用电负荷峰值下，实现了企业不拉闸、居民不限电。

　　——长沙企业享受到更多政策红利。“千人帮千企百日大行动”解决企业各类问题3580个，向上争取和兑现惠企政策资金32.5亿元，促成银企意向融资118亿元，企业发展的信心更足、预期更稳。

　　——长沙智能制造走在全国前列。国家级智能制造试点示范企业（项目）总数达27个，居全国省会城市第一；湖南湘江新区智能驾驶测试区获工信部授牌，成功跻身“国家队”；第三届中国军民两用技术创新应用大赛金奖数居全国第一。

　　——长沙内陆开放新高地加速崛起。获批国家跨境电商综合试验区、市场采购贸易方式试点、文化出口基地；进出口总额增长40%，增速居中部省会城市首位。

　　一年来，着重抓了七个方面的工作：

　　一是聚焦“三大攻坚战”。防范化解重大风险攻坚战有序推进。加快政府债务置换，严格预算管理，“停缓调撤”项目735个、涉及总投资2932亿元，完成99家政府融资平台公司“撤并转”。综合运用PPP、专项债等形式破解融资瓶颈，向民间资本推介项目25个、总投资675亿元，保障了重大项目顺利推进。开展非法集资专项整治，关停非法融资平台45家。精准脱贫攻坚战持续发力。统筹市内精准脱贫、市外对口帮扶，全市建档立卡贫困户脱贫32506人，3.07万贫困人口实现饮水安全；推进龙山县对口帮扶和7个国家级贫困县“携手奔小康”，实施扶贫项目283个、投入2.83亿元。污染防治攻坚战取得阶段性成效。持续推进中央、省环保督察反馈问题和中央生态环保督察“回头看”交办问题整改，平稳有序退出绿心保护区工业企业516家；率先全国推行盾构土环保处置，城区规模以上施工工地实现扬尘在线监测全覆盖；新增纯电动公交车957辆，查处高排放机动车3万余辆，完成餐饮单位油烟净化9650家、家庭餐厨油烟净化8.8万户。整改问题排污口25个，新改建乡镇、工业园区污水处理厂15座，岳麓尾水工程通水，24处黑臭水体治理通过国家验收，成为全国首批城区黑臭水体治理达标城市。人工造林6.77万亩，退耕还林还湿6285亩，森林覆盖率提升到54.95%，绿色成为长沙的鲜明底色。

　　二是聚焦实体经济。产业链建设成效明显。深入开展“产业项目建设年”活动，重大产业项目完成投资2480.6亿元，其中22条产业链项目完成投资963.2亿元。先进储能材料等产业链产值增幅达到30%以上。现代服务业加快发展，规模以上服务业单位达1667家，增长23.8%，服务业对经济增长的贡献率达58%，提高1.3个百分点。长沙银行成为全省首家上市银行；全域旅游加快推进，宁乡市获评全国最具魅力文化旅游城市；智能制造峰会、移动互联网岳麓峰会、糖酒会、农博会等展会成功举办。项目建设全速推进。省“五个100”项目任务全面完成。伟创力一期、杉杉能源正极材料、日写触控传感器等项目建成投产；中联智慧产业城、蓝思科技黄花基地、健康生态科技产业园等“百亿级”项目加快建设，腾讯、京东无人车总部、航天科技、德国大陆、天岳碳化硅等一批战略投资项目和企业成功落户，新签约投资2亿元以上项目178个。园区活力加快释放。向园区下放市级审批权限28项，开展相对集中行政许可权改革试点，基本实现园区事园区办。园区规模工业增加值增长10.9%，长沙高新区在全国高新区综合评价中居第12位，长沙经开区跻身全国经开区20强，宁乡经开区、望城经开区、岳麓高新区分别获批国家绿色园区、国家新型工业化产业示范基地和国家检验检测高技术服务业集聚区。创新能力持续提升。国家自主创新示范区政策覆盖省级以上园区，新建院士专家工作站17家，研究与试验发展经费支出占地区生产总值的比重达2.6%。高新技术企业突破2300家，高新技术产业总产值增长9.26%。获批创建国家知识产权强市，万人有效发明专利拥有量29.6件，居中部省会之首，知识产权保护中心率先全国运营，湖南湘江新区专利大数据平台上线运行。获批知识产权军民融合国家试点，军民融合产业总产值突破1000亿元。浏阳市获批创建全国首批创新型县（市）。大力创建质量强市示范城市，参与制定国际标准7项、国家标准62项。

　　三是聚焦改革开放。重点改革统筹推进。28项国家级、18项省级、12项市级改革年度任务全面完成。“最多跑一次”改革成效明显，“四个一”“四个办”强力实施，“互联网+政务服务”平台成功上线，市本级“最多跑一次”事项、“一次也不跑”事项分别达1401项、208项，企业开办时间缩短到4个工作日；宁乡市“互联网+政务服务”改革受到国务院通报表扬。“证照分离”改革纵深推进，取消、简化、优化行政许可99项，市场活力不断增强，市场主体总数达92.42万户。国资国企改革加快推进，分层分类管理体制进一步完善，市属国企资产突破万亿元；驻长央企、省企职工家属区“三供一业”分离移交试点走在全国前列。农村改革试点顺利推进，浏阳市农村土地制度改革、雨花区集体产权制度改革等国家试点全面完成，望城区城乡发展一体化改革深入实施，长沙县农村综合性改革获批国家试点。对外开放步伐加快。开放通道有效拓展，新开通国际客货运航线8条，完成黄花机场T1航站楼改造；中欧班列（长沙）线路增至11条；霞凝新港三期建设有序推进，铜官港试运营。开放主体不断壮大，进出口总额5000万美元以上外贸企业达50家，增长30%，跨境电商试点企业达51家。开放政策日益完善，开放型经济“2+4”政策加快落地，外贸扩规模、提质量“1+10”政策制定实施。交流合作不断深化。新增国际友好城市3个，圆满承办“一带一路”青年创意与遗产论坛、落实《南海各方行为宣言》高官会、国际商事法律论坛、中部（长沙）进口博览会，长沙与世界精彩互动。成功举办长江中游城市群省会城市会商会、长株潭城市群一体化发展联席会、湘赣边区域开放合作主题联席会，区域合作更加密切。

　　四是聚焦重大平台。湖南湘江新区“三区一高地”格局加快形成，华谊兄弟电影小镇等项目建成，宜家荟聚、卓伯根总部等项目加快推进，中国长城、湖南天玥等重大项目成功落户；集聚国家“千人计划”专家20名、创新创业服务平台550个，研究与试验发展经费支出占地区生产总值的比重达5.2%。岳麓山国家大学科技城建设成效明显，麓山南路、后湖综合整治完成；中南大学科技园总部建成运营，引进企业102家；桃子湖高校双创街、西湖互联网58小镇等加快建设，后湖艺术园开园。马栏山视频文创产业园建设步入“快车道”，获批首家国家级广播电视产业园，创梦天地等企业区域总部纷纷落户，注册落地企业171家。临空经济示范区配套设施建设提速，黄花综保区注册企业284家，实现进出口额25亿美元。高铁会展新城建设加快推进，长沙国际会议中心、城际空间站等加快建设，举办品牌会展53个，带动商品交易864.4亿元。湖南金融中心集聚能力持续增强，入驻金融机构及配套企业300家，总注册资本360亿元；湘江基金小镇基金管理规模达2025亿元，荣获特色小镇发展潜力奖。南部片区规划纲要公开发布，铺排重大项目21个，人力资源产业园开园。

　　五是聚焦乡村振兴。农村“五治”强力实施。完成改厕2.8万座，浏阳市率先全省实现旱厕清零；全市农村垃圾分类减量50%以上，长沙县农村垃圾分类处置体系全面建立；清理整治“一户多宅”“空心房”1.92万户306万平方米；16万个小微水体纳入“河长制”责任体系；移风易俗成为新时尚，涌现出望城区静慎村、长沙县金桥村、浏阳市秧田村等精神文明建设先进典型。新型村级集体经济来势良好。实施“四型”集体经济项目448个，带动村级集体经济创收5.3亿元、农民增收3.8亿元，消除集体经济薄弱村283个，长沙县率先全省消除集体经济薄弱村。现代农业加速发展。种养大户、家庭农场、农民合作社、农业龙头企业等新型农业经营主体分别新增1374家、676家、854家、108家，分别增长11.6 %、17.8 %、7.9%和136%；调整农作物种植结构29.55万亩，形成30个千亩、4个万亩特色种植片区；“三品一标”农产品615个；实现农产品加工业销售收入2319亿元。农村基础设施加快完善。新建农村公路1227公里，提质改造农村公路700公里。清淤山塘2.8万亩，修复水毁工程3.9万处，整修小河坝823座。白石洞水库加快建设，椒花水利枢纽纳入国家重大水利项目库。

　　六是聚焦城市品质。城市规划不断优化。全域划定“三区三线”，建立全要素空间管控体系；形成新一轮城市总规修编初步成果，编制村庄规划400个；“多规合一”在线审批平台正式运行。城市建设加快推进。长益常铁路、蒙华铁路长沙段加快建设；岳望高速、岳宁大道全线通车；西二环拓改、橘子洲大桥提质改造、洞井路和曙光路跨京广铁路桥等工程竣工。地下综合管廊试点通过国家验收；新增城市公园84个；新建绿色建筑1151万平方米；长沙国金中心建成运营，刷新城市新高度。修复损毁堤防53公里，敢胜垸等15个城市防洪圈加快建设。城市运营提质提效。健全市、区两级城市建设投入责任与收益分配机制。划定城市有机更新单元24个，潮宗街、白果园、太平街等街区和红星、雅塘等片区有机更新抓紧推进，改造老旧社区50个。城市管理持续加强。“数字城管”与网格化管理有机融合，城市管理下沉到627个网格，基本实现问题发现在网格、解决在网格。主城区清扫保洁实现全覆盖，拆除违法建筑73万平方米；城区生活垃圾减量13.37%，市生活垃圾深度综合处理项目正式运行。“大数据+靶向治理”交通管理经验在全国推介。

　　七是聚焦社会事业。就业服务不断优化，城镇新增就业14.49万人，城镇登记失业率控制在3%以内。社会保障加快完善，企业退休人员养老金、城乡居民基础养老金分别实现14连调、13连调；80周岁及以上老年人高龄津贴实现普惠提标；新增城乡居家养老服务中心31家、养老床位2956张；城乡低保提高至每人每月550元，困难残疾人生活补贴、重度残疾人护理补贴分别达到每人每月120元、80元。教育事业持续发展，建成义务教育标准化学校303所，新增学位7.6万个；完成小区配套幼儿园移交188所，全市普惠性幼儿园占比达80%；关停无证办学机构1656家；完成卫生职院新校区主体工程。健康长沙深入推进，市级医院提质提档工程进展顺利，新改扩建乡镇、村（社区）卫生服务机构36个、医联体79个，成功创建全国基层中医药工作先进市；城乡居民医保补助提高至每人每年490元。文体事业日益繁荣，建设示范性村（社区）综合文化服务中心100个，新华联铜官窑古镇、田汉文化园、铜官窑博物馆、非遗展示馆建成开放，《乡村国是》获第七届鲁迅文学奖；成功举办国际马拉松赛、国际名校赛艇挑战赛，实现省运会金牌、总分“双第一”。新青少年宫建成开放。成功举办纪念刘少奇同志诞辰120周年活动。其他事业协调推进，社科普及、科普服务、供销、移民、粮食、职工维权、妇女儿童等工作取得新进展，民族宗教、港澳台侨、机关事务、公积金、地方志、档案、气象、水文、地震、慈善、红十字等工作发挥新作用。

　　一年来，各民主党派、工商联、无党派人士围绕中心、服务大局，参政议政、建言献策，有力推动了长沙发展。实行市领导领办民生建议和重点提案，办理人大代表建议357件、政协提案408件，办复率100%。办结中央巡视组移交信访件1223件；依法审查规范性文件643件、政府合同320件，公共法律服务站实现村级全覆盖，成功调解矛盾纠纷4.2万件；完成公共资源交易4783宗；完成审计项目82个，挽回损失3.57亿元。支持军队改革，加强国防动员、后备力量及人民防空建设，落实双拥优抚安置，军政军民更加团结。

　　各位代表！“时光荏苒，岁月留香”。时间蕴藏着伟力，奋斗见证着辉煌。改革开放40年，“心忧天下、敢为人先”的长沙人民风雨同舟、奋楫争先，用智慧和汗水创造了无愧于时代的业绩。40年来，长沙经济总量从不足20亿元先后跨越百亿、千亿台阶，步入万亿俱乐部，跃居全国省会城市前列；40年来，长沙城市建成区面积扩大8倍，由“五一路时代”迈入“湘江时代”，开拓了“一江两岸、比翼齐飞”的城市新格局，跻身国内新一线城市；40年来，长沙城镇、农村居民收入（扣除物价增长因素）分别增长20.1倍和25.7倍，城镇居民人均居住面积由3.97平方米增加到40.4平方米，老百姓获得感越来越强，幸福成为长沙的鲜明标识。“历尽天华成此景，人间万事出艰辛”。这些成绩的取得，是省委、省政府和市委正确领导的结果，是市人大及其常委会和市政协监督支持的结果，是全市上下团结拼搏的结果。在此，我代表市人民政府向全市各族人民，向各民主党派、工商联、无党派人士和人民团体，向驻长部队、武警官兵、政法干警、消防救援和民兵预备役人员，向所有关心、支持和参与长沙发展的社会各界人士，表示诚挚的感谢和崇高的敬意！

　　“风雨多经志弥坚，关山初度路犹长”。我们清醒地看到，发展中还存在一些问题和不足：传统制造业智能化改造亟待加快，新兴产业仍处于成长期，有全国影响力的产业集群、龙头企业不多，规模以上工业增加值增速、税收占财政收入比重未达到预期目标，外向型经济和现代服务业的短板明显，产业结构与高质量发展要求仍存在差距；中小微企业、个体户生产经营面临困难，融资、用地、用工等问题比较突出，生产要素配置与做强市场主体要求仍存在差距；农业产业发展碎片化较为严重，“散、小、弱”问题突出，与现代农业的发展要求仍存在差距；城市管理精细化、数字化水平有待提升，教育、医疗、养老、食品安全等公共服务有待优化，与市民对美好生活的需要仍存在差距；一些干部不敢担当、不想作为、慢作为的问题较为明显，与求真务实、一抓到底的要求仍存在差距。对此，我们将高度重视，采取有效措施加以解决。

　　二、2019年形势与任务

　　今年是新中国成立70周年，是全面建成小康社会关键之年。长沙发展与全国、全省一样，仍然处于重要战略机遇期，相比之下，外部环境比去年难，但内部条件比去年好。从大局看，世界面临百年未有之大变局，经济形势错综复杂，不确定不稳定因素增多，危和机同生并存；我国经济运行稳中有变、变中有忧，面临下行压力，但国家宏观政策强化逆周期调节，继续实施积极的财政政策和稳健的货币政策，供给侧结构性改革突出“巩固、增强、提升、畅通”，政策环境总体向好。从长沙看，机遇与挑战并存，机遇是长沙产业链成长壮大，开放型经济加速崛起，营商环境不断优化，高质量发展的基础更加夯实；挑战是投资增长乏力、消费结构亟待优化，转型升级压力较大，正处于爬坡过坎、不进则退的关键阶段。我们必须保持战略定力、聚焦主要矛盾，真抓实干、稳扎稳打，化压力为动力、变不利为有利，走在改革最前列，抢占发展制高点。

　　今年政府工作总体要求是：以习近平新时代中国特色社会主义思想为指导，认真贯彻党的十九大精神和习近平总书记关于湖南工作的重要指示精神，坚持稳中求进工作总基调，坚持新发展理念，坚持推动高质量发展，坚持以供给侧结构性改革为主线，坚持深化市场化改革、扩大高水平开放，坚持创新引领开放崛起，深入推进现代化长沙和“三个中心”建设，继续打好“三大攻坚战”，着力激发市场主体活力，加快传统产业转型、创新成果转化、新旧动能转换、政府职能转变，全面实施改革开放提效、城市品质提升、乡村振兴提速、民生保障提标，进一步稳就业、稳金融、稳外贸、稳外资、稳投资、稳预期，保持经济持续健康发展和社会大局稳定，以优异成绩庆祝中华人民共和国成立70周年。

　　全市经济社会发展主要预期目标是：地区生产总值增长8%；固定资产投资增长10%；社会消费品零售总额增长10%；地方一般公共预算收入增长8%；研究与试验发展经费支出占地区生产总值比重2.8%以上；产业项目投资占固定资产投资比重45%以上，制造业（含高技术服务业）投资占固定资产投资比重达35%；单位地区生产总值能耗下降2%，税收占财政收入比重、减排任务完成省定指标；全体居民人均可支配收入增长8%，城镇登记失业率控制在4%以内，居民消费价格指数103左右。

　　为实现上述目标，工作中必须牢牢把握以下五个方面：

　　一是突出省会担当。把握省会城市这一最基本的定位，牢记使命、不负重托，深度融入“一带一路”、长江经济带等国家战略，发挥“一带一部”首位城市优势，推动中央各项战略部署在长沙落地生根，带头落实省委、省政府决策部署，在引领全省发展特别是长株潭一体化发展中强化省会担当，在服务全省大局上发挥省会作用，当好创新引领开放崛起的排头兵。

　　二是突出智能制造。继续推进“产业项目建设年”活动，坚持以智能制造为统领，以产业链建设为着力点，加快动能转换，落实“三智一自主”产业布局，推进智能化改造扩面升级，打造更多智能化企业和产品，推动制造业高质量发展，争当智能制造领跑者。

　　三是突出平台支撑。充分发挥湖南湘江新区等国家级战略平台优势，持续提升区域综合竞争力；建好用好园区平台、创新平台、开放平台，推动政策制定精准化、运营服务专业化、资源集聚高效化，打造制度高地、创新高地、开放高地、人才高地，加速将潜在竞争力转化为现实生产力。

　　四是突出环境优化。开展“营商环境优化年”活动，把“安不安全、方不方便、赚不赚钱、清不清廉”作为重要标尺，以公平公正的法治环境为引领，实施流程再造、效能革命，加快建设更高效的政务环境、更开放的投资环境、更优良的生产环境、更便利的贸易环境，让政务运行透明公开、企业发展预期稳定、群众办事方便快捷，全力打造国际一流营商环境。

　　五是突出民生改善。坚持保基本兜底线，推动公共资源向基层延伸、向农村覆盖、向弱势群体倾斜，进一步解决好就业、上学、养老、医疗、出行、居住等群众切身利益问题，织密扎牢社会保障网，让全市人民享有更多的获得感、幸福感、安全感。

　　三、2019年工作重点

　　突出抓好以下十个方面的工作：

　　（一）强力推进现代产业发展。坚持先进制造业与现代服务业深度融合，大力发展实体经济，加快建设现代化产业体系。以智能制造引领制造业高质量发展。紧盯产业链建设，聚焦产业高端和终端，培育产业联盟，完善“两图两库两池两报告”，实现产业投资子基金对产业链的全覆盖，加快形成更为完善的产业链生态体系。着力培育新动能、发展新经济，突出数字经济、智能经济、绿色经济、创意经济、共享经济“五大新经济形态”，强化新一代人工智能、新能源、功能材料、信息终端、生物基因、视频等“六大技术”攻关，打造智能网联汽车、工业互联网、智慧城市、智慧医疗、移动支付、分享经济、区块链“七大应用场景”；争创国家级网络安全产业园区。加快推进企业智能化改造，力争智能制造试点企业达到600家。坚持质量引领，推进中国计量院长沙基地建设，打造国家检验检测高技术服务业集聚区；支持中国一乡一品产业促进中心建设，着力打造国家级产业融合发展平台。推进高新、高效、智慧、绿色园区建设，强化产业定位，实现市、区（县、市）两级行政审批权限对省级以上园区应放尽放，完善以亩均效益和项目履约率为重点的考核体系。持续推进“五个100”项目建设，强化招商市级统筹，引进更多龙头型、基地型、旗舰型项目和500强企业、行业领军企业；抓好8英寸集成电路装备验证工艺线、大陆集团中央电子工厂、群显科技显示屏等项目，建成投产华智生物、格力智能装备、豪恩声学等项目。以消费升级助推现代服务业发展。围绕满足最终需求，加快现代服务业集聚区建设，服务业增加值增长9%以上。大力发展现代商贸，高品质规划建设现代化集中商业区，提质改造五一、红星等商圈和金星路等商业街区；加快发展电子商务，改善消费环境，培育网络消费、定制消费、体验消费等新热点，推动消费升级，推广应用和包支付，打造中国“移动支付第三城”。提质发展现代金融，深入实施企业上市行动计划，提高上市公司质量；积极发展普惠金融，加大对中小微企业等薄弱领域的金融支持。创新发展现代物流，加快供应链体系试点建设，提升物流标准化、信息化水平，社会物流总费用占地区生产总值比率降至14.8%；支持红星市场申报进出口水果查验平台，加快海吉星农副产品市场、大河西农产品物流中心等建设发展。着力发展现代会展，引进3个以上国际高端展会落户，办好首届中非经贸博览会、中国国际工程机械展等展会，继续办好移动互联网岳麓峰会、智能制造峰会、“一带一路”青年创意与遗产论坛、国际商事法律论坛等活动。集聚发展现代楼宇，打造一批特色楼宇、品质楼宇。融合发展现代文旅，支持耀邦故里、洋湖湿地创建5A级景区，实现华强文化产业园开园；推进国家文化出口基地和市戏剧艺术中心建设，高水平建设长沙交响乐团，高标准打造世界媒体艺术之都，用文化演绎长沙元素、讲述长沙故事、彰显长沙力量。

　　（二）强力推进“三大攻坚战”。针对突出问题，强化精准施策，打好重点战役。坚决打好防范化解重大风险攻坚战。坚持控增量、调结构、守底线，进一步规范政府举债行为，严格债务限额管理和预算管理，完成23家平台公司“撤并转”任务。坚持化解政府债务与扩大有效投资相统筹，更好地发挥专项债券对稳投资、扩内需、补短板的作用，突出增加产业发展和基础设施有效投资，带动社会投资。严厉打击非法集资等违法违规金融活动。坚决打好精准脱贫攻坚战。紧扣“两不愁、三保障”标准，确保84个省定贫困村稳定退出，建档立卡贫困户脱贫6000人以上，减少和防止贫困人口返贫。突出产业和就业扶贫，实施产业扶贫项目500个，新增建档立卡贫困劳动力转移就业1000人。对收入水平略高于建档立卡贫困户的群体制定支持政策。加强对口帮扶，助推龙山县精准脱贫，与7个国家级贫困县携手奔小康。坚决打好污染防治攻坚战。抓好中央生态环保督察“回头看”反馈问题整改。严格绿心地区项目准入，完成剩余10个工业企业（项目）退出，帮助企业制定环境治理解决方案，推进绿心地区生态修复。打赢蓝天保卫战，深入推进“六控十禁”，加强工业废气、扬尘、机动车排放等污染防治，加大科技治霾力度，完成社区餐厨油烟治理6万户以上，空气质量优良率78%以上。打好碧水保卫战，纵深推进湘江保护与治理“一号重点工程”，严格落实“河长制”，持续抓好“一江六河”截污治污，深入开展河湖“清四乱”等专项整治，加强黑臭水体治理和乡镇污水处理厂运营管理，扩容提标金霞等5个污水处理厂，建成运营苏圫垸等污水处理厂。推进净土保卫战，强化土壤污染管控和修复，加快黑麋峰环保主题公园建设。实施静音保卫战，重点加强交通噪音治理。环境就是民生，青山就是美丽，蓝天就是幸福，让长沙处处呈现天蓝、地绿、水清的生态美景。

　　（三）强力推进改革开放。坚持以改革推动开放、以开放倒逼改革，实现改革开放互动并进。深化以供给侧结构性改革为主线的各项改革。深入落实供给侧结构性改革“1+7”政策，巩固“三去一降一补”成果。全面完成国家级、省级改革年度任务和市县两级政府机构改革。深入推进“最多跑一次”改革，“一次办”“网上办”比率均达95%以上，证明材料再精简30%以上。全域推行“证照分离”改革，实现市场主体“一照一码走天下”。深化国资国企改革，稳妥推进股权多元化和混合所有制改革，做强做优做大国有资本。深化财税体制改革，强化全口径预算管理、预算绩效评价及刚性约束，提高财政资源配置效率。深化户籍制度改革，完善单位和社区集体户制度，抓好已在城镇就业农业转移人口的落户工作。全面推进自然资源综合管理制度改革，实施全域自然资源统一确权登记。稳妥推进统计体制、供销合作社、粮食收储制度等改革。积极推进全方位对外开放。加快临空经济示范区、黄花综保区、跨境电商综试区融合发展，大力引进和培育龙头企业，力争跨境电商交易额突破10亿美元。发挥国家市场采购贸易方式试点、外贸转型升级基地作用，加快培育贸易新业态新模式，推动开放型经济指标高速增长。积极申建霞凝铁路一类口岸、国际航空邮件经转口岸，加快霞凝新港三期、铜官港二期建设，推进中欧班列（长沙）、“五定”班列（轮）平稳高效运营，新开5条以上国际客货运航线，打造多式联运新优势。加快智慧口岸建设，全面推广国际贸易“单一窗口”，提升通关效率、降低通关成本。进一步完善国际标志标识，加快建设国际学校、社区和医疗机构，推动更多外国政商机构、国际组织分支机构落户长沙。务实推进区域协同发展。加快推进长株潭一体化，带头开好联席会议，带头落实2019年行动计划，带头办好“三干两轨”“一卡通”等20项合作实事；依托南部片区和湖南湘江新区，双向对接株洲、湘潭发展。深度对接粤港澳大湾区，主动融入泛珠三角区域创新发展，积极承接产业转移和科技成果转化；推动设立国家级湘赣开放合作试验区。主动对接长江经济带，推进长沙港与上海港、城陵矶港深度合作，强化与长三角、长江中游城市群在贸易、产业等领域合作。深度融入“一带一路”，支持企业重点拓展东欧、东南亚市场，积极走进非洲、对接非洲、深耕非洲，精心办好埃塞—湖南工业园等境外经贸园区，积极筹建乌干达—湖南产业园，大力发展“走出去”联盟，扩大产品进出口和国际产能合作，引导长沙企业抱团出海，打造扬帆远航的“联合舰队”。站在新的起点，我们将把改革的大旗举得更高，把开放的步子迈得更大，将改革开放进行到底。

　　（四）强力推进民营经济发展。真心实意、真金白银支持民营企业，推动民营经济走向更加广阔的舞台。支持民营企业做强做优。设立中小企业发展专项资金，发展更多“专精特新”科技型“小巨人”企业；支持企业“入规”、上市、“升高”，在做大规模、直接融资、高新技术发展上取得新突破；实现市场主体突破100万户。争创民营经济改革创新示范区。促进民营企业减税降负。全面落实阶段性降低社会保险缴费费率、养老保险过渡费率试点、小型微利企业减半征收企业所得税等政策；支持符合条件的民营企业天然气用户转供改直供，落实一般工商业用电降价政策，将价格红利传导到终端用户。缓解民营企业融资难题。引导设立民营企业债券融资支持工具，加快组建融资担保公司；鼓励金融机构落实无还本续贷制度，提高中长期贷款比例；落实国家小微企业融资担保业务降费奖补等政策，把更多金融“活水”引向小微企业。优化民营企业发展环境。全面清理和废止阻碍民营经济市场主体参与公平竞争的政策文件，保障民营企业平等地位。进一步压缩审批时限，企业开办、不动产登记、工业建设项目施工许可证审批周期分别减少到3个、5个、45个工作日内。引导民营企业参与养老服务机构、文化体育设施建设运营，鼓励民营企业参与国有企业重大投资、成果转化和资产整合项目。大力支持建筑业发展。保障民营企业合法权益。建立民营企业家与涉企部门负责人常态化沟通制度，鼓励聘请企业家作为经济顾问。营造法治化制度环境，保护企业家人身和财产安全，倡导诚信守法经营，弘扬优秀企业家精神，让长沙成为企业家的投资旺地、成长胜地、生活福地。

　　（五）强力推进创新驱动发展。以创新型城市建设为统揽，加快国家自主创新示范区建设，以科技创新带动全面创新，打造发展新引擎。夯实创新基础。坚持技术研发平台重点聚集技术孵化、园区平台重点聚集成果转化，以湘江智谷、中国V谷、种业硅谷、高新麓谷等为依托，围绕产业链建设，培育引进更多高水平研发机构、优秀创新团队、高端创新人才，创建岳麓山实验室、国家生物种业技术创新中心。推进军民深度融合，加快省军民融合科技创新产业园、知识产权军民融合试点建设，引导创建军民融合企业技术中心，积极创建国家军民融合创新示范区，实现军民融合产业总产值增长20%以上。继续实施高新技术企业“强基工程”行动，高新技术企业达2500家，高新技术产业增加值占地区生产总值比重35%以上。优化创新环境。健全以需求为导向、企业为主体的产学研一体化创新机制，激励企业推动产业链协同创新和产业孵化集聚创新，成立市级科技创新基金，扩大科技创新券发放对象和适用范围。深入推进知识产权强市创建，充分发挥知识产权保护中心作用，完善新兴领域、新兴业态知识产权保护制度。吸纳创新人才。全面推进“芙蓉人才行动计划”“人才政策22条”落地落细，释放政策叠加效应。发挥海外引才联络站等平台作用，紧盯企业、园区和创新研发平台的人才需求，突出高端人才、青年人才、工匠人才，在引进培育、创新创业、服务保障等方面持续发力，把长沙打造成创新的蓝海、创业的沃土、创客的乐园。

　　（六）强力推进湖南湘江新区和重大片区建设。强化发展规划引领、重大项目支撑、管理体制创新，打造经济发展的引爆点、彰显品质长沙的新优势。突出提升湖南湘江新区引领示范作用。以高端制造业、高技术服务业为重点，着力打造全国智能网联汽车创新和产业化高地，推动新区走在人工智能产业发展前列。大力推进洋湖总部经济区、月亮岛文旅新城、长沙高铁西城等片区建设，加快人和未来、和光文化产业基地建设，实现湘江欢乐城、恒大文化旅游城开园。统筹河西基础设施配套，加快麓景路南延线、长望路西延线和湖南奥林匹克体育中心建设，实现雷锋水质净化厂（一期）竣工。铺排重大项目450个，完成投资1200亿元以上。加快推进岳麓山国家大学科技城产城深度融合。以“一圈一廊一街一镇一区”为重点，加快发展科创经济，新引进项目100个以上，完成投资50亿元以上。提质改造阜埠河路、牌楼路，加快建设中建国际产业园、后湖之芯，设立大科城知识产权交易服务中心，引导更多高端创新资源进入中南大学研发总部等平台，向着世界一流大学城的目标迈进。高质高效抓好马栏山视频文创产业园建设。落实“部省共建”协议，加快打造具有国际影响力的视频文创产业园区。推进马栏山（月湖）文创基地、马栏山公园、鸭子铺片区主次干道等综合配套建设；突出产业引领，瞄准行业龙头，新引进视频文创企业200家，完成投资50亿元以上。办好中国新媒体大会。对标全国一流提升临空经济示范区发展水平。做大做强保税加工、口岸贸易、跨境电商、金融服务四大平台，加快跨境电商产业园、药品物流基地、全球海鲜长沙集散中心和商品分拨中心建设，集聚发展临空偏好型产业，新引进项目200个，完成投资50亿元以上。推进龙峰大道、机场北辅道等道路建设，加快构建以机场为核心的综合交通体系。着力推进高铁会展新城发展。主攻高铁商务区、会展功能区，提质会展产业生态系统，新引进项目200个，完成投资100亿元以上，全力打造国际中高端会议承办地、现代服务业聚集新区。加快长沙国际会议中心、国际交流区等项目建设，完善会议中心周边景观、高铁片区产业、公共服务等项目配套。加快湖南金融中心建设。进一步完善基础设施和公共服务，新引进金融科技领军企业、大型金融机构、基金机构不少于100家，完成投资45亿元以上。

　　推进科技普惠金融国家试点，争取金融数据交易中心、大数据实验室等公共平台项目落户。加快湘江基金小镇建设，大力发展产业风险投资、股权投资等基金，构建“湘江基金群”，办好湘江金融峰会。实质性推动南部片区建设。统筹片区重大基础设施、产业发展、公共服务、生态环境建设，新引进项目100个以上，完成投资50亿元以上，打造融城发展生态圈。有序推进牛角塘、中信新城等区域建设，加快实施韶山南路、红旗路南延线和湘雅五医院等项目，重点发展文化旅游、健康养老、地理信息等产业，创建国家级人力资源服务产业园，将南部片区加快建设成为两型发展引领区、融合发展核心区、创新发展示范区。

　　（七）强力推进品位品质品牌城市建设。提升城市规划建设管理水平，从旧有的经营城市理念中走出来，用精明增长理念指导城市发展。发挥规划引领作用。高质量编制国土空间规划，进一步优化“三区三线”，做到一本规划、一张蓝图覆盖全域。有序疏解核心城区功能，将金阳新城、金洲新城打造成长沙“一体两翼”格局的重要支撑。完善城市承载功能。加快实施原107国道城市化改造和湘雅路过江通道建设，实现湘府路快改工程建成通车；推进黄花机场空铁枢纽、渝长厦高铁、长沙西站铁路枢纽建设；加快长益高速复线建设，启动宁韶、江干高速建设；进一步完善轨道线网规划方案，推进5、6号线建设，确保4号线载客运营、3号线通车试运行。完成七水厂新建、一水厂提质改造、望城水厂扩建。持续推进“630攻坚”，确保500千伏浏阳变等项目投产、1000千伏长沙特高压等项目开工。加强工业互联网、物联网等新型基础设施建设。提升城市管理水平。实施文明行为促进条例，打造全国文明城市“升级版”。完善城市管理执法体制，提升管理科学化、精细化、智能化水平。全面推进生活垃圾分类减量，社区覆盖率达到100%。启动市生活垃圾深度综合处理二期、固废处理场灰渣填埋场建设，加快浏阳、宁乡生活垃圾焚烧项目前期工作。全面完成城区公厕、垃圾中转站和住宅小区垃圾站、环卫车辆新能源改造任务，在橘子洲景区打造全国首个新能源全智能化环卫保洁示范基地。加强智慧交通平台建设，规范社会停车场管理，推进换电站、充电桩建设。推进城市有机更新。开展城市双修，加强湿地保护，加快井塘示范公园建设，新增城区绿地300公顷以上，推进国家生态园林城市创建。落实“四增两减”，持续推进棚改新三年行动计划。提质改造老旧小区50个以上。精心打造白沙液街、潮宗街、文庙坪等历史街区，保护贾谊故居等历史遗存，让一条条老街古巷留住长沙记忆，传承历史文脉。

　　（八）强力推进乡村振兴战略。突出乡村环境整治和新型村级集体经济发展，加快建设产业兴旺、生态宜居、乡风文明、治理有效、生活富裕的现代化乡村。发展现代都市农业。深化农业供给侧结构性改革，进一步调优农业产业结构。突出“一县一特”，大力发展都市休闲、生态观光、特色高效等新型农业，打通一二三产业链条，加快培育蔬菜、花猪、花卉苗木、绿茶、油茶等单一农产品产值过100亿元、50亿元的特色产业，支持浏阳国家级田园综合体试点、宁乡国家农村产业融合发展示范园、长沙县春华现代农业示范园建设，农产品加工业销售收入突破2500亿元。强化农业品牌建设，新增“三品一标”50个以上。推进“优质粮食”和“菜篮子”工程建设，增加优质绿色农产品供给。推动农民持续增收。着力将小农户引进现代农业发展大格局，重点培育种养大户、家庭农场、农民合作社、农业龙头企业，新增新型农业经营主体2000家以上。实施农民增收三年行动，探索农村劳务经纪人制度，带动农村劳动力转移就业；鼓励农民就近创办农业企业，支持农民采取合作、参股等方式发展产业经营项目，提升创业质量；突出盘活空闲农房和农地资源，推动民宿经济发展；农村居民人均可支配收入增长8%以上。改善农村人居环境。大力推进农村“五治”，实现农村旱厕“清零”，强化新建农房规划管控，巩固提升移风易俗成效。强力推进畜禽粪污整治，确保资源化利用率超过80%。加强农村公路安防设施建设。继续推进农村饮水安全巩固提升工程，启动浏阳清江等6个水厂扩容提质改造。加快城乡防洪保护圈建设，推进大众垸防洪综合整治，完成湘江东岸防洪综合改造和乔口、沩丰等大中型水闸除险加固，启动椒花水利枢纽工程。开展乡村振兴示范创建行动，打造示范乡镇20个、示范村200个。关心支持边远乡镇发展，加大基础设施建设投入，让边远乡镇不“边缘”。壮大新型村级集体经济。大力发展“四型”集体经济，深化集体林权制度改革，继续保持土地承包关系稳定，用活耕地、激活集体建设用地，引导新增村级土地合作社100家、土地流转面积占耕地总面积比重达到55%以上，让农业更有干头、农民更有赚头、农村更有奔头。

　　（九）强力推进民生福祉改善。坚持以人民为中心，办好民生实事，提供更多更好的公共服务产品。更好地推动幼有所育、学有所教。加快学前教育普及普惠安全优质发展，提高普惠性幼儿园比例，继续推进住宅小区配套幼儿园移交；加快义务教育阶段学校建设，创建中小学标准化学校130所，加强乡镇寄宿制学校建设，建成培智特殊教育学校；推进职业教育校企深度合作；完成长沙卫生职院新校区建设，加快湖南信息职院新校区、长沙幼儿师范高等专科学校建设；支持长沙学院等市属高校和“双一流”大学高质量发展。推进民办学校分类管理改革，规范校外托管和培训机构管理；推行中小学教师“县管校聘”改革，加强教师队伍建设。更好地推动病有所医、健有所保。深化医药卫生体制改革，落实分级诊疗，加强家庭医生签约服务；推进新一轮基层医疗卫生机构建设，加强县级医院建设和乡村医生、全科医生培养，传承发展中医药事业，提升基本医疗和公共卫生服务水平；推进“健康长沙”PPP项目、“智慧医疗”项目和医联体建设，完成市四医院滨水新城院区建设，启动市妇幼、口腔等医院分院建设前期工作，支持爱尔眼科发展，打造“眼健康”产业之都。推动群众体育和竞技体育全面发展，建设区域性全民健身中心，启动长沙国际体育中心建设，办好中日韩青少年运动会等赛事，打造体育名城。更好地推动劳有所得、弱有所扶。坚持就业优先，突出做好高校毕业生、农民工、退役军人、城镇困难人员等群体就业工作，城镇新增就业13万人；加强劳动保障监察执法，维护职工权益。统筹各项救助资源，加大低保兜底对象和特困人员保障力度，解决因残致贫家庭突出困难。提升社会福利、慈善、红十字等公益服务水平。推进儿童友好型城市创建，让孩子们更加安全、自由、快乐地成长。更好地推动老有所养、老有所乐。探索建立长期护理保险制度，发展老龄事业和产业，推动社区医疗与养老资源结合，鼓励社会力量参与社区居家养老。加快老年友好型城市建设，丰富老年群体的精神文化生活，办好老年大学，让老人的晚年更加多姿多彩。更好地推动住有所居、居有所安。落实“房子是用来住的、不是用来炒的”要求，稳房价、稳地价、稳预期，严格规范房地产开发和中介企业经营行为，引导住房租赁市场发展，努力让刚需人群有房可住、买得起房。规范小区物业管理，支持老旧住宅加装电梯。办好10件民生实事。一是高质量完成“一圈两场三道”建设任务，完善智慧管理平台功能，提升“15分钟生活圈”服务水平，将温暖工程、幸福工程送到老百姓“家门口”。二是新改扩建公办幼儿园30所。三是全面启动区县（市）社会福利中心建设，建成社区居家养老服务中心30家、敬老院照料护理区30所。四是新改建农村公路1000公里，完成自然村通水泥沥青公路省定目标任务。五是实现全市行政村（社区）小微水体管护全覆盖，建设小微水体管护试点片区20个。六是建设标准化村（社区）综合文化中心100个。七是免费实施新生儿多种遗传代谢疾病筛查50000个，对全市0—18岁儿童青少年眼及视力筛查保健，建立健康电子档案。八是新建全民健身工程600处、智慧健身驿站20个，新增公园70个，其中体育公园2个。九是提质改造小餐饮“透明厨房”4000家。十是完成城镇棚户区改造和老城区有机更新13500户。同时，全面完成省定民生实事任务。

　　（十）强力推进社会治理。加快打造共建共治共享的社会治理新格局，让城市安全运行、社会安定有序。创新基层社会治理。深化街道管理体制改革，推进资源配置下沉，将街道工作重心转向优化公共服务。推进城市网格化管理，实现城市基层社会治理全网覆盖、人网联动。创新“三社联动”服务，规范发展社会组织，加强专业社工和志愿者队伍建设。夯实农村基层治理基础，农村社区建设覆盖率达85%。强化信息数据支撑，加快推进二维码标准地址建设，筑牢智慧长沙建设基石。加大市场监管力度。理顺监管体制，推动工作重心从事前审批转向事中事后监管，实现“双随机”监管方式全覆盖、检查结果全公开。严格食品药品监管，实现食品安全追溯体系全覆盖，建成市民满意的国家食品安全示范城市。全面开展质量提升行动，完善质量标准体系，提升产品、服务、工程和环境质量，建设质量强市。提升应急管理能力。建立健全灾害事故防范救援机制，加快构建统一指挥、反应灵敏、协同有序、运转高效的应急管理体系，牢牢守住安全生产底线，加强道路交通等重点行业领域安全监管，坚决防范和遏制重特大事故发生。全面完成12个中心消防站和11个应急取水设施建设，完善相关配套，确保年内投入使用。提高气象、水文等精准预警预报能力，实现气象预报精准到乡镇。深化平安长沙建设。加快“星城快警”“一村一辅警” 建设，建立“互联网+群防群治”体系，健全市场主体、社会力量广泛参与的网状治理模式，打造重点区域快速反应圈。持续深入开展“扫黑除恶”等专项斗争，依法严厉打击违法犯罪行为。创新信访服务模式，推动信访积案化解常态化。开展人民陪审员选任，推进司法民主。完善人民调解、行政调解、司法调解衔接联动机制，提升社会矛盾预防化解能力。持续深化“七五”普法规划实施，加快创建全国公共法律服务体系建设示范城市。打好禁毒人民战争。推进全国禁毒示范城市创建，健全禁毒责任体系，强化重点人员、物品、场所管控，做实社区戒毒社区康复工作，探索妇联组织参与禁毒工作、青少年毒品预防教育新模式，保持打击毒品违法犯罪高压态势，让毒品远离每个家庭，让公平正义的阳光温暖每个人的心田。

　　加强国防动员、后备力量建设；支持军队军事政策制度改革，突出抓好民兵调整改革任务落实，完善退役军人管理服务保障；深化普及全民国防教育，落实双拥优抚工作，争创全国双拥模范城“七连冠”。推动全国少数民族流动人口管理服务示范城市创建。扎实开展第四次全国经济普查。全面加强国家安全、人防、对台、机关事务、地方志、档案、地震、移民安置等工作。积极支持工会、共青团、妇联、科协、文联、侨联、社科联等群团组织工作。

　　四、切实加强政府自身建设

　　不断满足人民群众对美好生活的向往，是政府工作的追求目标。必须始终坚持党的领导，把依宪施政、依法行政贯穿政府工作全过程，主动接受人大法律监督、工作监督，自觉接受政协民主监督，全面履行政府职能，加快建设法治政府。

　　坚持担当干事。为官避事平生耻，重任千钧惟担当。政府工作的关键是落实，推进落实的根本在担当。视使命如生命。政府工作人员绝对忠诚地担负起建设现代化长沙的重大使命，积极投身改革发展主战场、攻坚克难第一线、服务群众最前沿，在履职尽责中体现作为、彰显价值。市政府班子成员带头为改革担当、为项目担当、为发展担当，带头领任务、解难题、促发展，确保重点工作统筹推进、分管领域亮点纷呈。凭实绩论英雄。坚持以发展排座次、以项目比能力，把项目建设作为“赛马场”，持续掀起新一轮大抓项目、抓大项目热潮。政府各级各部门围绕大局、紧扣中心、对标任务，强化政策执行、狠抓工作落实，确保事事有着落、件件都办成。为担当者担当。健全、落实正向激励机制、容错纠错机制、澄清关怀机制，保护改革者，鼓励探索者，宽容失误者，旗帜鲜明地为敢于担当的干部担当、为敢于负责的干部负责，让干部放下包袱、甩开膀子干事创业、建功立业。

　　坚持高效干事。起跑就是冲刺，开局就是决战。大力推进政府“效能革命”。让管理服务更快捷。坚持以大数据、云平台、互联网为支撑，纵深推进“最多跑一次”改革，建设全市智慧城市运行中心，升级“多规合一”信息平台，构建标准统一、四级联动的政务服务平台，加快线上线下融合，推动信息共享、流程优化、全市通办。让群众办事更方便。推动“四个办”落实落细，将“就近办”事项全面融入“15分钟生活圈”，让老百姓办事不出社区、企业办事不出园区；提高“网上办”比率，拓展政务服务网上应用和移动应用，做到让数据多跑路、群众少跑腿；加大“减证办”力度，除政府公布的证明材料清单外，一律不再要求提供其他材料；探索“帮代办”服务，扩大办理事项范围，不断提升市民和市场主体满意度。让政务运行更透明。全面深化财政预决算、公共资源配置等重点领域信息公开，加强环境保护、突发事件、重大疫情等重点事项信息发布。大力推广基层政务公开标准化规范化试点，稳步推进政府数据共享开放，让阳光照亮权力运行的每个角落。

　　坚持务实干事。厚植公仆情怀，弘扬实干精神，带着感情、带着责任为群众和企业解难题、办好事。体察实情。持续开展企业帮扶活动，经济部门班子成员每月走访10家以上重点企业，推动政策入企惠企。严格落实“三走访三签字”，真正把脱贫攻坚工作做到困难群众的心坎上。更加重视基层、关爱基层干部，改善基层工作条件，大力精简会议报表材料，统筹规范督查检查考核，为基层减负减压。办好实事。坚持新增财力向民生支出倾斜，全力办好普惠性、基础性、兜底性民生工作，突出抓好以“一圈两场三道”为重点的民生实事，确保每件实事落地落细、落实到位，持续提升群众幸福感。务求实效。认真执行人大及其常委会的各项决定、决议，竭诚办好人大代表建议、意见和政协提案。严厉整肃庸政懒政怠政，坚决破除形式主义、官僚主义，改进文风会风，政府系统规范性文件及会议均减少10%以上。厉行节约，部门预算的项目经费压减5％以上。着力提升政府系统干部素质和执行力，让“马上就办”蔚然成风、“真抓实干”落地生根。

　　坚持干净干事。按照“严字当头、全面从严、一严到底”的要求，以“永远在路上”的执着纵深推进政府系统党风廉政建设，确保干部清正、政府清廉。严守廉洁底线。把党风廉政建设作为底线工作，与政府日常工作、重点工作同步研究、同步部署、同步落实，层层压实责任、传导压力。把纪律和规矩挺在前面，广泛开展法纪教育和警示教育，引导政府工作人员不断增强廉洁自律意识，干干净净为人民做事。不越法纪红线。落实“三重一大”事项决策机制，加强行政规范性文件监督管理，推进合法性审查全覆盖；统筹推进综合行政执法改革，做到公正执法、文明执法。聚焦落实中央八项规定精神、打好三大攻坚战、优化营商环境等重点，严查政府系统违纪违规案件，扎实开展“提篮子”“红包礼金”等专项整治，坚决整治群众身边腐败和作风问题。筑牢监督防线。紧盯公共权力、公共资金、公共工程、公共资源和资产，切实加强审计监督，支持开展派驻监督、巡查监督、监察监督。认真落实人大执法检查、视察、专题询问和政协协商意见，主动接受社会和舆论监督，确保人民赋予的权力始终为人民服务。

　　各位代表，奋斗成就梦想，实干开创未来。让我们更加紧密团结在以习近平同志为核心的党中央周围，在省委、省政府和市委的坚强领导下，不忘初心、牢记使命，以“咬定青山不放松”的定力，以“不待扬鞭自奋蹄”的干劲，全心全意为人民谋幸福，尽力尽责为长沙谋发展，奋力谱写建设现代化长沙的壮丽篇章，以高质量发展的优异成绩迎接中华人民共和国成立70周年！
